# Supplementary material for: Splice-Junction-Based Mapping of Alternative Isoforms in the Human Proteome
Source: Cell Rep. Author manuscript; Available in PMC 2020 Jan 15. (PMC6961840; doi:10.1016/j.celrep.2019.11.026)
Supplement: 2 [file NIHMS1546469-supplement-2.zip › DF1/PXD010154/Testis/Testis_36_PDLIM7_SHAFSHAPTLPCPPALPGCVSAQASAPAADPPRY.pdf]

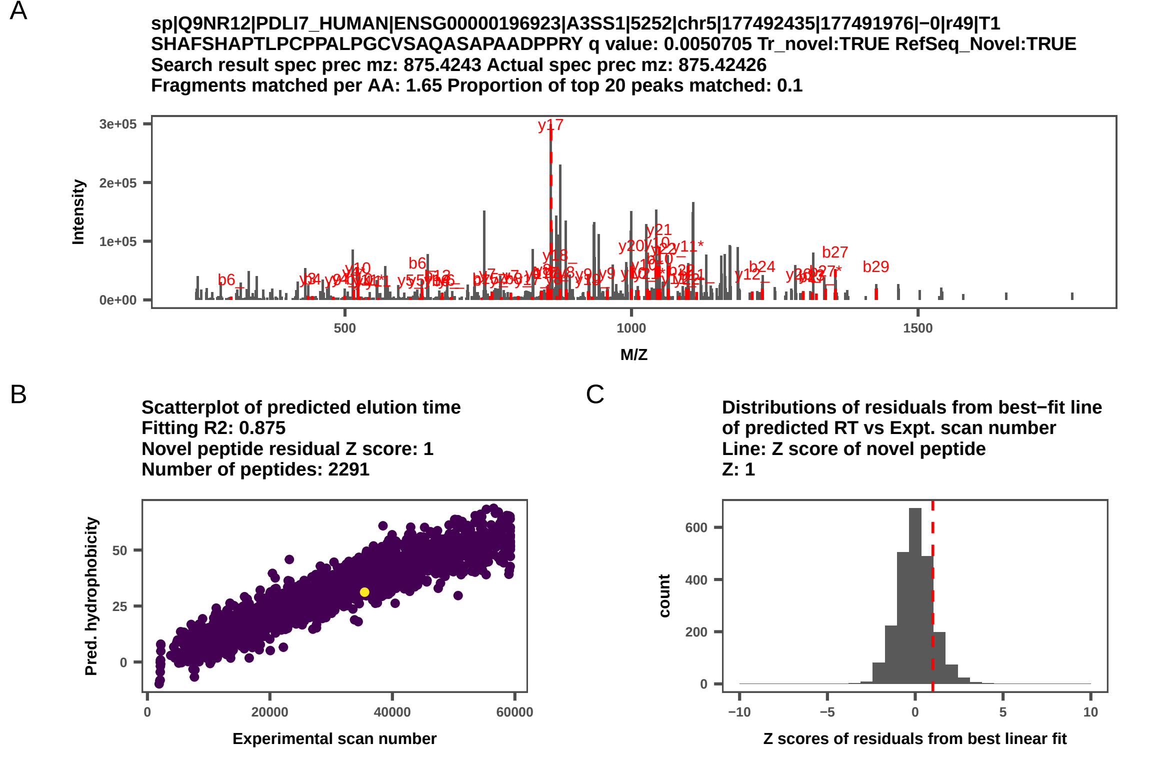
A
sp|Q9NR12|PDLI7_HUMAN|ENSG00000196923|A3SS1|5252|chr5|177492435|177491976|−0|r49|T1
SHAFSHAPTLPCPPALPGCVSAQASAPAADPPRY q value: 0.0050705 Tr_novel:TRUE RefSeq_Novel:TRUE
Search result spec prec mz: 875.4243 Actual spec prec mz: 875.42426
Fragments matched per AA: 1.65 Proportion of top 20 peaks matched: 0.1
Intensity
3e+05
2e+05
1e+05
0e+00
y17
y21
b27
b24
b29
b6
M/Z
500
1000
1500
B
Scatterplot of predicted elution time
Fitting R2: 0.875
Novel peptide residual Z score: 1
Number of peptides: 2291
Pred. hydrophobicity
50
25
0
0
20000
40000
60000
Experimental scan number
C
Distributions of residuals from best−fit line
of predicted RT vs Expt. scan number
Line: Z score of novel peptide
Z: 1
count
600
400
200
0
−10
−5
0
5
10
Z scores of residuals from best linear fit
